# Recognizing myorhythmia 4 months after stroke – A teaching video

**DOI:** 10.1016/j.prdoa.2022.100141

**Published:** 2022-03-14

**Authors:** Sebastian Finkener, Tobias Piroth, Magdalena Högg, Stephan Rüegg, Krassen Nedeltchev, Julien F. Bally, Markus Gschwind

**Affiliations:** a Department of Neurology, Cantonal Hospital Aarau, Aarau, Switzerland; b Department of Neurorehabilitation, REHAB Basel, Basel, Switzerland; c Department of Neurology, University Hospital Basel, Basel, Switzerland; d Service of Neurology, Department of Clinical Neurosciences, University Hospital of Lausanne and Lausanne University, Switzerland; e Service of Neurology, Department of Clinical Neurosciences, University Hospital of Geneva and Geneva University, Switzerland

**Keywords:** Myorhythmia, Stroke, Guillain-Mollaret triangle, Red nucleus, Thalamus, Superior cerebellar peduncle

## Abstract

In this case study with video and neurophysiology, we describe a rare case of hemimyorhythmia occurring 4 months after a stroke with bilateral affection of the thalamus and right superior cerebellar peduncle (Guillain-Mollaret-triangle). This case and especially the video with the clinical and EMG presentation of a synchronous rhythmic pattern at 3,1 Hz makes an important educational contribution to the recognition of myorhythmia and discussed differential diagnoses.

Myorythmia is a hyperkinetic involuntary movement disorder characterized by slow (1–4 Hz) rhythmic movements of the cranial or limb muscles [1]. Its etiologies are very diverse, the most frequent ones being brainstem/thalamic infarctions or hematomas, Whipple’s disease among other posterior fossa infections, NMDAR-encephalitis and cerebellar degeneration due to chronic alcoholism [2].

We report the case of a 47-year-old woman who was admitted to our hospital for long-term electroencephalographic monitoring due to intermittent rhythmic movements of the right hemibody of unclear origin. Four months prior she suffered a vertebrobasilar stroke (Fig. 1A) due to extravascular coiling of an iatrogenic basilary tip arterial laceration during a transsphenoidal clival meningioma resection.

**Fig. 1:**
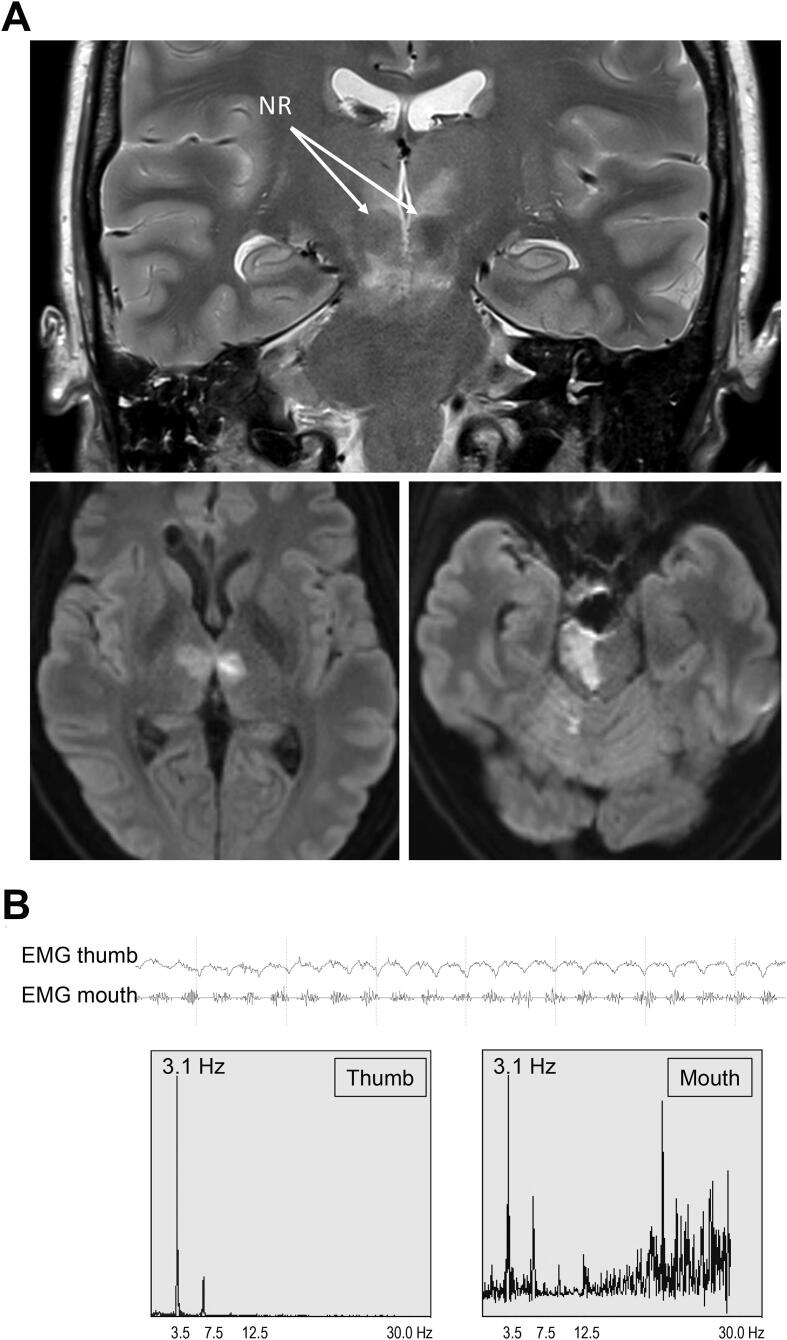
A. Brain MRI at day 1 after vertebrobasilar stroke. Top: The T2-weighted image shows a hyperintense stroke lesion involving both thalami and bilateral nucleus ruber (NR), and descending further down to the right superior cerebellar peduncle. Bottom: The diffusion-weighted images display acute ischemic hyperintense lesions involving parts of the Guillan-Mollaret-Triangle connecting the contralateral red nucleus in the midbrain, the contralateral inferior olivary nucleus in the medulla, and the ispilateral dentate nucleus in the cerebellum via the central tegmental tract and the inferior and superior cerebellar bundles. In our case no hypertrophy of the olivary nucleus was seen. Image displayed in radiological convention. B. Upper part: Surface-EMG of the right hand’s thumb and the right angle of the mouth showing synchronous bursts of motor unit potentials. Lower part: The spectrograms of the time–frequency analysis of both EMG-signals using a fast Fourrier-transform reveals the same dominant frequency of 3.1 Hz for both signals.

Clinically she presented with spastic tetraparesis, accentuated on the left side and persistent altered level of consciousness. She showed episodes of rhythmic, slow movements affecting the right face (perinasal region) and jaw, the palate and tongue, as well as the distal right upper- and lower extremities. These movements developed progressively 4 months after the stroke, would persist at rest, were stress-triggered (patient positioning, oropharyngeal suctioning, emotional discussion, etc.) and mostly disappeared during sleep. EEG monitoring did not reveal any associated epileptic discharges (Video – published with patient’s consent). Surface EMG of the right M. abductor pollicis brevis and right M. orbicularis ori showed perfectly synchronous rhythmic burts, lasting approximately 200 ms with a frequency of 3.1 Hz (Fig. 1B). Medication trials with levetiracetam, brivaracetam, lacosamid, carbamazepin, oxcarbazepin, phenytoin, primidon, levodopa/benserazide and piracetam, showed no clinical improvement. Low dose of valproic acid and clonazepam finally could attenuate the symptomatology. Tetrabenazine was not tried.

The clinical picture here is consistent with myorhythmia affecting the right hemibody. Other phenomenologies to be considered in the differential of myorhythmia include parkinsonian tremor, dystonic tremor, Holmes tremor and other forms of rhythmic myoclonus, for instance myoclonic jerks occurring in the setting of epilepsia partialis continua, which can all be rather well differentiated clinically and electrophysiologically (Table 1). Pathogenesis of myorhythmia points towards a lesion in the Guillain-Mollaret-triangle (connections between contralateral nucleus ruber, contralateral nucleus olivaris and ipsilateral nucleus dentatus) [1], [3], [4], although some published cases showed an involvement of the sole thalamus [2]. Our case demonstrated bilateral lesions in both the thalamus and nucleus ruber, as well as the right superior cerebellar peduncle, consitent with myorhythmia in the right hemibody. In our case the movements were synchronous, confirming a single pacemaker activity; asynchronous movements of different body parts have also been reported in myorhythmia [1], [4].

**Table 1:** Differential diagnosis of Myorhythmia and clinical/electrophysiological distinctive features, put together according to different authors [1], [2], [4].

Differential phenomenological diagnosis of myorhythmia	
Holmes tremor	- No cranial involvement - Kinetic tremor has higher amplitude than postural tremor and postural tremor has higher amplitude than rest tremor (whereas the amplitude remains constant in myorhythmia)
Parkinson tremor	- Slightly higher frequency (4–5 Hz) than myorhythmia (0.5–4 Hz) - “Pill Rolling” tremor - Levodopa responsiveness - Other parkinsonian signs and symptoms
Dystonic tremor	- Action > rest - More irregular or pseudo-rhythmic - Usually higher frequency (4–7 Hz) - Presence of Geste antagoniste - “Null point”, where the tremor subsides
Myoclonic jerks due to epilepsia partialis continua	- Shorter burst duration (25–75 msec), whereas myorhythmia averages 200 ms - Generally visible EEG jerk-locked discharges

Myorhythmia is a rare but clinically and electrophysiologically well-defined movement disorder. Burst duration on surface EMG is usually around 200 ms [1], as in our case. In contrast, segmental myoclonus can have an even longer EMG burst duration, up to 500 ms [1], [5], [6], which distinguishes it from cortical myoclonus which is shorter than 50 ms, and from cortical-subcortical myoclonus which is shorter than 100 ms. Frequency most often stands between 1 and 4 Hz, but a wider range is admitted by some authors, from 0.2 to 8 Hz [5], [6]. Myorhythmia can persist in low-stages of sleep but usually disappears during deep sleep [7].

Myorhythmia is important to identify, as some of its etiologies are treatable (for instance Whipple’s disease or NMDAR-encephalitis), with poor outcomes if left untreated. Myorhythmia of these etiologies is treated via the primary disease (antimicrobials passing the blood–brain barrier, and immonutherapy, respectively). In myorhythmia of structural etiology (mostly post-stroke, as in our patient) treatment response is usually limited. Among the various antiepileptic and neuroleptic agents that can be tried [1], substances with a high GABAergic mechanism such as valproic acid or clonacepam might show better efficacy. Moreover, both valproic acid or clonacepam also provide some mood stabilisation and affective control, given the fact that our patient's myorythmia episodes seemed to worsen in the context of emotional stress.

## Disclosures

The other authors have nothing to declare related to this project.

## Author contribution

SF conceived the study, drafted and revised the manuscript, TP diagnosed the patient, conceived the study, drafted and revised the manuscript, MH revised the manuscript, KN contributed important knowledge, drafted and revised the manuscript, SR conceived the study, contributed important knowledge, drafted and revised the manuscript, JFB conceived the study, contributed important knowledge, drafted and revised the manuscript, MG diagnosed the patient conceived and supervised the study, contributed important knowledge, drafted and revised the manuscript.

## Declaration of Competing Interest

The authors declare that they have no known competing financial interests or personal relationships that could have appeared to influence the work reported in this paper.

## Appendix A Supplementary data

Supplementary video 1 The patient displayed synchronous rythmic movements at about 3 Hz, affecting the right side of the perinasal and palatal region, jaw and tongue, as well as distal upper and lower extremities.
